# Supplementary material for: Predation and fragmentation portrayed in the statistical structure of prey time series
Source: BMC Ecol. 2009 May 6;9:10. doi: 10.1186/1472-6785-9-10 (PMC2689204; doi:10.1186/1472-6785-9-10)
Supplement: Additional file 2 — Voles and related classes ODDox Documentation. ODDox documentation of the agent-based model (ALMaSS) applied by Hendrichsen et al. The documentation is started by activating main.html. [file 1472-6785-9-10-S2.zip › Vole_ODDox/functions_func_0x70.html]

ALMaSS ODDox: Class Members - Functions

- Main Page
- Related Pages
- Classes
- Files

- Alphabetical List
- Class List
- Class Hierarchy
- Class Members

- All
- Functions
- Variables

- a
- b
- c
- d
- e
- f
- g
- h
- i
- k
- l
- m
- n
- o
- p
- r
- s
- t
- u
- v
- w
- ~

### - p -

- PesticideTrialControl()
  : PesticideTrialControl- PesticideTrialToxicControl()
    : PesticideTrialToxicControl- PesticideTrialTreatment()
      : PesticideTrialTreatment- PigsAreOut()
        : Farm- PigsAreOutForced()
          : Farm- PigsOut()
            : Farm- Population\_Manager()
              : Population\_Manager- PrintChromosome()
                : GeneticMaterial
                , GeneticMaterial1616- Probe()
                  : Population\_Manager- probe\_data()
                    : probe\_data- ProbeFileInput()
                      : Population\_Manager- ProbeReport()
                        : Population\_Manager- ProductApplication0()
                          : Farm- ProductApplication1()
                            : Farm

---

Generated on Thu Jan 22 14:13:45 2009 for ALMaSS ODDox by 
 1.5.6
